# In Vitro Activity of Omadacycline and Comparator Antibiotics against Extended-Spectrum Beta-Lactamase-Producing *Escherichia coli* and *Klebsiella pneumoniae* Urinary Isolates

**DOI:** 10.3390/antibiotics12060953

**Published:** 2023-05-24

**Authors:** Tyler J. Stone, Abdullah Kilic, John C. Williamson, Elizabeth L. Palavecino

**Affiliations:** 1 Department of Pharmacy, Atrium Health Wake Forest Baptist, Winston-Salem, NC 27157, USA; tyler.stone23@gmail.com; 2 Department of Pathology, Wake Forest School of Medicine, Winston-Salem, NC 27157, USA; akilic@wakehealth.edu (A.K.); epalave@wakehealth.edu (E.L.P.); 3 Department of Internal Medicine, Section on Infectious Diseases, Wake Forest School of Medicine, Winston-Salem, NC 27157, USA

**Keywords:** omadacycline, extended-spectrum beta-lactamase (ESBL), urinary tract infections, *Escherichia coli*, *Klebsiella pneumoniae*

## Abstract

Limited oral antibiotic options exist for urinary tract infections (UTI) caused by ESBL-producing Enterobacterales. The aim of the study was to evaluate in vitro activity of omadacycline and comparator antibiotics against clinical ESBL-producing and non-ESBL-producing *E. coli* and *K. pneumoniae* urinary isolates. 102 isolates each of *E. coli* and *K. pneumoniae* were collected from clinical urine specimens in 2019. By design, an equal number of each species were included that tested positive and negative for ESBL production. Omadacycline MICs were determined using gradient test strips and compared to MICs of comparator antibiotics as determined by an automated broth microdilution system. Isolates were considered susceptible to omadacycline if the MIC was ≤4 µg/mL for each species. 54.9% of all ESBL-producing isolates were susceptible to omadacycline, but better susceptibility was observed for ESBL-producing *E. coli* (74.5%). Omadacycline MICs were 2–4 fold lower for *E. coli* and *K. pneumoniae* strains not producing ESBL. The omadacycline MIC 50 and 90 values were 4 and 16 µg/mL, respectively, for all isolates studied. 74.5% of all isolates were considered susceptible to omadacycline. MICs were generally lower for *E. coli* strains with MIC 50 and 90 values of 4 and 8 µg/mL, respectively (87.3% susceptible), compared with *K. pneumoniae*. Overall, the most active agents were omadacycline and nitrofurantoin, while other comparator antibiotics were less active. Omadacycline represents a promising oral antibiotic for treating UTI caused by ESBL-producing *E. coli*, particularly when resistance limits other oral options. Prospective, controlled clinical trials are needed to validate these in vitro results.

## 1. Introduction

Urinary tract infection (UTI) is one of the most common causes of bacterial infection requiring antibiotic therapy [1,2]. Despite the fact that many different microorganisms have been identified as causative pathogens in UTIs, *Escherichia coli* and *Klebsiella pneumoniae* are among those most frequently implicated [3]. UTI recurrence rates are often high and antimicrobial resistance has been increasing against currently available oral antibiotics [4]. Furthermore, extended-spectrum beta-lactamase (ESBL)-producing Enterobacterales have been increasingly identified [5,6]. UTI caused by ESBL-producing Enterobacterales often leave medical providers with a limited number of effective treatment options, especially orally administered antibiotics [7,8]. The World Health Organization has published a list of target organisms with priority to help guide the development of new antibiotics [9]. In this list, third generation cephalosporin-resistant Enterobacterales, e.g., ESBL-producers, are considered a critical priority.

Omadacycline is the first novel agent in the class of aminomethylcyclines. Omadacycline has been shown to have in vitro activity against Enterobacterales isolates resistant to traditional tetracycline antibiotics [10]. Oral and intravenous (IV) omadacycline is approved by the US Food and Drug Administration (FDA) for acute bacterial skin and skin structure infections and community acquired bacterial pneumonia in adults. Its mechanism of action is similar to other tetracycline antibiotics, inhibiting protein synthesis via 16S rRNA component of the 30S subunit of the bacterial ribosome. However, omadacycline has a two-fold higher binding affinity for the 30S ribosome than tetracycline [11]. Although the documented spectrum of in vitro activity for omadacycline includes ESBL-producing Enterobacterales, this agent has not been FDA-approved for treatment of UTI, the most common infection caused by ESBL-producing Enterobacterales [7]. The aim of the study was to evaluate in vitro activity of omadacycline and comparator antibiotics against clinical ESBL-producing and non-ESBL producing *E coli* and *Klebsiella pneumoniae* isolates recovered from urine specimens.

## 2. Materials and Methods

A total of 102 *E. coli* and 102 *K. pneumoniae* non-duplicate strains were isolated consecutively from clinical urine specimens of adult patients submitted to the clinical microbiology lab of Atrium Health Wake Forest Baptist between March and October 2019. By design, an equal number of each species were included that tested positive and negative for ESBL production as determined by MicroScan WalkAway system (Beckman Coulter West Sacramento, CA, USA). The samples were processed for quantitative urine culture by inoculating a 10 µL urine sample onto 5% sheep blood and MacConkey agar plates and incubated at 35 °C for 18–24 h according to standard protocols. Organisms were identified to the species level by matrix assisted laser desorption ionization-time of flight mass spectrometry (MALDI-TOF MS) (Bruker Daltonics, Billerica, MA, USA).

All isolates were tested in triplicate for omadacycline susceptibility and the minimum inhibitory concentrations (MIC) were determined using MIC gradient test strips (Liofilchem, Inc., Waltham, MA, USA) according to the manufacturer’s instructions. In addition, five oral antibiotics commonly prescribed for the treatment of UTIs (tetracycline, amoxicillin-clavulanate, trimethoprim-sulfamethoxazole, nitrofurantoin, and ciprofloxacin) were tested by broth microdilution using the MicroScan WalkAway system. MIC results for these comparator agents were interpreted in concordance with Clinical and Laboratory Standards Institute (CLSI) M100 guidelines [12]. In the absence of omadacycline CLSI breakpoints for *E. coli*, FDA breakpoints of *K. pneumoniae* were applied [13]. For the purpose of this study, all isolates were considered susceptible to omadacycline if the MIC was ≤4 µg/mL. MIC 50 and 90 values were calculated for categories of isolates according to species and ESBL-production. Chi Square test was performed to compare proportions within and between categories, e.g., omadacycline vs. comparator antibiotics, *E. coli* vs. *K. pneumoniae*. This study was approved by the Institutional Review Board of Wake Forest University Health Sciences, including waiver of the requirement to obtain informed consent.

## 3. Results

Susceptibility results, including MIC 50, MIC 90, MIC range, modal MIC, and percent susceptible, are summarized in Table 1. For all ESBL-producing isolates, the omadacycline MIC 50/90 was 4/>32 with 54.9% susceptible, which was significantly lower than non-ESBL-producing isolates (91.2%, *p* < 0.001). Omadacycline was second only to nitrofurantoin (71.6% susceptible) among ESBL-producing isolates (*p* = 0.014). Tetracycline, ciprofloxacin, and trimethoprim-sulfamethoxazole performed poorly against ESBL-producing isolates as the percent susceptible for these was no greater than 26.5% (*p* < 0.001 vs omadacycline). Omadacycline was generally more active (about one dilution lower MIC) against *E. coli* than *K. pneumoniae* (87.3% vs. 61.8% susceptible, *p* < 0.001). 100% of non-ESBL-producing *E. coli* were susceptible to omadacycline. The omadacycline MIC 50/90 values were 2/4 and 4/16 µg/mL for non-ESBL-producing and ESBL-producing *E. coli*, respectively, and 4/8 and 8/>32 for non-ESBL-producing and ESBL-producing *K. pneumoniae*, respectively. For all isolates studied (*N* = 204), the omadacycline MIC 50 and 90 values were 4 and 16 µg/mL, respectively, with 74.5% susceptible.

**Figure 1:**
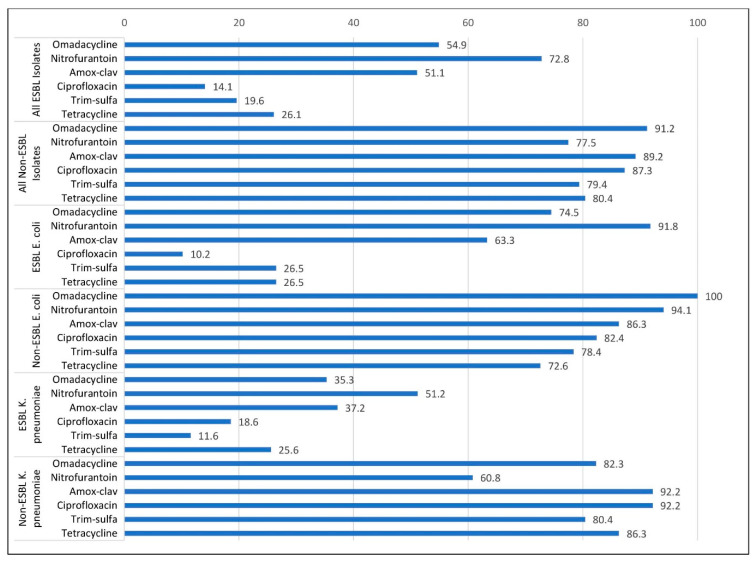
Percent susceptible of omadacycline and comparator antibiotics.

For comparison purposes, Figure 1 is a visual representation of the percent susceptible for all antibiotics within the categories of isolates. For ESBL-producing isolates, the susceptibility rate to tetracycline, amoxicillin-clavulanate, trimethoprim-sulfamethoxazole, nitrofurantoin, and ciprofloxacin varied, ranging from 10.2% to 91.8% for *E. coli* and 11.6% to 51.2% for *K. pneumoniae* (Table 1). Omadacycline and nitrofurantoin were the most active agents tested against urinary isolates for both ESBL-producing and non-ESBL-producing *E. coli* and *K. pneumoniae* included in this study. Nitrofurantoin performed well against ESBL-producing *E. coli* and was the only antibiotic that performed better than omadacycline in this category (91.8% vs. 74.5% susceptible, *p* = 0.001). Compared to ESBL-producing *E. coli*, the percent susceptible for ESBL-producing *K. pneumoniae* was lower for all antibiotics tested. Overall, the least active agents against ESBL-producing isolates were trimethoprim-sulfamethoxazole and ciprofloxacin. Omadacycline displayed lower MICs compared to tetracycline regardless of ESBL production. For *E. coli* isolates, the percent with MIC ≤ 4 µg/mL was 87.3% for omadacycline compared with 47.3% for tetracycline (*p* < 0.001).

## 4. Discussion

UTIs affect 150 million patients globally per year and are the leading cause of all infection-related outpatient visits in the United States [14]. Among oral antibiotics, nitrofurantoin and trimethoprim-sulfamethoxazole have been recommended as first-line empiric therapies for acute uncomplicated cystitis [3]. Fluoroquinolones, such as ciprofloxacin, are recommended as alternatives for cystitis due to their broad spectrum of activity and risk for escalating resistance [3]. Resistance within the limited number of oral antibiotic options for UTI is making the choice of therapy complicated.

UTIs caused by Enterobacterales harboring antimicrobial resistance has increased rapidly and can lead to life threating urosepsis. The increasing prevalence of ESBL production among Enterobacterales seriously compromises the activity of many oral antibiotics commonly prescribed for UTI [7]. ESBL-producing Enterobacterales are associated with increased length of hospital stay and mortality [15,16]. Nitrofurantoin remains sufficiently active against ESBL-producing *E. coli*, but does not provide reliable activity against ESBL-producing *K. pneumoniae* strains [17], and is not recommended for infections involving upper urinary tract, i.e., pyelonephritis. Furthermore, co-harboring of resistance mechanisms has been observed in ESBL-producing *E. coli* isolates, further limiting oral antibiotic options [7]. Therefore, new antibiotic options are needed for treating UTIs caused by ESBL-producing Enterobacterales.

Omadacycline as a treatment of UTI has been investigated in two phase 2 studies of uncomplicated cystitis [18] and acute pyelonephritis [19], respectively. In these studies, omadacycline failed to demonstrate non-inferiority versus nitrofurantoin for cystitis and levofloxacin for pyelonephritis. However, different dosing regimens of omadacycline were evaluated in these studies. Results analyzed according to the different dosing regimens suggest the possibility of a dose-response relationship. A trend for increasing rates of clinical success was demonstrated with doses that would result in higher omadacycline exposures. A potential explanation for this effect was described by Pagano and colleagues [20]. Microbiologic activity of omadacycline was shown to be reduced in urine compared with standard culture media. This study also characterized the inhibitory effect that magnesium and calcium may have on the activity of omadacycline. Collectively, these results suggest that relatively aggressive dosing of omadacycline (e.g., 450 mg PO daily) may be necessary to achieve optimal outcomes in treating UTIs, or that omadacycline susceptibility breakpoints for treating UTIs should be lower than what is currently established. Lastly, it is important to note that the phase 2 UTI studies did not specifically evaluate efficacy of omadacycline against ESBL-producing organisms. This represents an opportunity for future research.

Omadacycline has potent in vitro activity against Enterobacterales, including strains that produce ESBL [21,22]. In our study, omadacycline demonstrated in vitro activity against 204 *E. coli* and *K. pneumoniae* isolates with 74.5% of isolates having an MIC ≤ 4 µg/mL, the susceptible breakpoint for *K. pneumoniae*. Pfaller et al. tested omadacycline and comparator agents against Enterobacterales urinary isolates collected in a surveillance program during 2010 and 2014. This study demonstrated that omadacycline was the most active agent tested against *E. coli* (MIC 50/90, 0.5/2 µg/mL with 99.4% of isolates inhibited at ≤4 µg/mL) and *Klebsiella species* (MIC 50/90, 2/4 µg/mL with 92.1% of isolates inhibited at ≤4 µg/mL) [23]. However, there was very low proportional representation by ESBL-producing isolates in the study. The percent susceptible in the study by Pfaller and colleagues was higher than that found in our study, for both the overall sample tested and the ESBL-producing isolates. The difference may be due to geographic location of isolates collected, variations in prevalence of ESBL-producing isolates, year of isolation, and microbiologic methodology (broth microdilution versus MIC gradient test strips). When comparing our results to those of other studies, the MIC 50 values of omadacycline against *E. coli* and *K. pneumoniae* isolates were marginally higher (4 vs. 1–2 µg/mL) but MIC 90 values were the same (16 µg/mL) [21,24].

Two distinct patterns of omadacycline in vitro activity were observed in our study. Omadacycline exhibited better activity against non-ESBL-producing isolates compared with ESBL-producing isolates. Omadacycline also demonstrated greater in vitro activity against *E. coli* than *K. pneumoniae*, including those that were ESBL-producing. Other investigators have also identified these patterns in omadacycline activity [23].

Our study is not without limitations. As this was an in vitro study of bacterial isolates collected in the laboratory, we did not confirm presence of urinary symptoms in the patients whose urine specimens were cultured in the lab, so we are not able to determine whether the isolates represent UTI or asymptomatic bacteriuria. Secondly, the method of susceptibility testing for omadacycline was different than that of the comparator antibiotics. This has the potential to result in slight differences in MICs, although major errors between the methods are not expected. Although the automated phenotypic method used in this study is a validated method to detect ESBL production, we did not perform genetic testing to identify specific ESBL genes in the isolates. However, for surveillance purposes, we have previously performed genetic testing of a collection of isolates within our health system that tested positive for ESBL production by the automated phenotypic method and found that >90% harbor CTX-M genes, the majority of which were CTX-M-15. Lastly, fosfomycin is another oral antibiotic option for UTI. Fosfomycin has microbiologic activity against ESBL-producing Enterobacterales, yet it was not included in the panel of the automated broth microdilution system used in the lab. For that reason, we are not able to make any comparisons between fosfomycin and the antibiotics evaluated in our study.

In conclusion, omadacycline exhibits promising in vitro antimicrobial activity against ESBL-producing *E. coli* and *K. pneumoniae* urinary isolates, including those that exhibit resistance to other oral antibiotics used to treat UTI. These data support the investigation of omadacycline as a potential oral option in the treatment of UTI caused by ESBL-producing *E. coli* and *K. pneumoniae*. In future studies of UTI, careful consideration should be given to the trial design to account for inhibitory effects of urine and the possibility of a dose-response relationship.

## Figures and Tables

**Table 1:** Activity of omadacycline and comparator antibiotics against *E. coli* and *K. pneumoniae*.

Microorganisms (n ^a^)	Antibiotics	MIC µg/mL					S%
		MIC_50_	MIC_90_	Minimum MIC	Maximum MIC	Modal MIC	
All ESBL-producing isolates (102)	Omadacycline	4	>32	0.25	>32	4	54.9 ^b^
	Tetracycline	>8	>8	≤4	>8	>8	26.1
	Amoxicillin-clavulanate	8/4	16/8	≤4/2	>16/8	16/8	51.1
	Trimethoprim-sulfamethoxazole	>2/38	>2/38	≤2/38	>2/38	>2/38	19.6
	Ciprofloxacin	>2	>2	≤1	>2	>2	14.1
	Nitrofurantoin	≤32	>64	≤32	>64	≤32	72.8
All non-ESBL-producing isolates (102)	Omadacycline	3	6	0.25	>32	4	91.2 ^b^
	Tetracycline	≤4	>8	≤4	>8	≤4	80.4
	Amoxicillin-clavulanate	≤4/2	16/8	≤4/2	>16/8	≤4/2	89.2
	Trimethoprim-sulfamethoxazole	≤2/38	>2/38	≤2/38	>2/38	≤2/38	79.4
	Ciprofloxacin	≤1	>2	≤1	>2	≤1	87.3
	Nitrofurantoin	≤32	64	≤32	>64	≤32	77.5
ESBL-Producing *E. coli* (51)	Omadacycline	4	16	0.25	>32	4	74.5 ^b^
	Tetracycline	>8	>8	≤4	>8	>8	26.5
	Amoxicillin-clavulanate	8/4	16/8	<4/2	16/8	16/8	63.3
	Trimethoprim-sulfamethoxazole	>2/38	>2/38	≤2/38	>2/38	>2/38	26.5
	Ciprofloxacin	>2	>2	≤1	>2	>2	10.2
	Nitrofurantoin	≤32	≤32	≤32	>64	≤32	91.8
Non-ESBL-Producing *E. coli* (51)	Omadacycline	2	4	1	8	3	100 ^b^
	Tetracycline	≤4	>8	≤4	>8	≤4	72.6
	Amoxicillin-clavulanate	<4/2	16/8	<4/2	>16/8	<4/2	86.3
	Trimethoprim-sulfamethoxazole	≤2/38	>2/38	≤2/38	>2/38	≤2/38	78.4
	Ciprofloxacin	≤1	>2	≤1	>2	≤1	82.4
	Nitrofurantoin	≤32	≤32	≤32	>64	≤32	94.1
ESBL-Producing *K. pneumoniae* (51)	Omadacycline	8	>32	2	>32	4	35.3
	Tetracycline	≤4	>8	>8	>8	>8	25.6
	Amoxicillin-clavulanate	<4/2	>16/8	<4/2	>16/8	16/8	37.2
	Trimethoprim-sulfamethoxazole	≤2/38	>2/38	≤2/38	>2/38	>2/38	11.6
	Ciprofloxacin	≤1	>2	≤1	>2	>2	18.6
	Nitrofurantoin	≤32	>64	≤32	>64	≤32	51.2
Non-ESBL-Producing *K. pneumoniae* (51)	Omadacycline	4	8	2	>32	4	82.3
	Tetracycline	≤4	8	≤4	>8	≤4	86.3
	Amoxicillin-clavulanate	<4/2	8/4	<4/2	>16/8	<4/2	92.2
	Trimethoprim-sulfamethoxazole	≤2/38	>2/38	≤2/38	>2/38	≤2/38	80.4
	Ciprofloxacin	≤1	>2	≤1	>2	≤1	92.2
	Nitrofurantoin	<32	>64	≤32	>64	≤32	60.8
All *E. coli* (102)	Omadacycline	4	8	0.25	>32	4	87.3 ^b^
	Tetracycline	≤4	>8	≤4	>8	≤4	47.3
	Amoxicillin-clavulanate	8/4	16/8	<4/2	>16/8	<4/2	73.1
	Trimethoprim-sulfamethoxazole	≤2/38	>2/38	≤2/38	>2/38	≤2/38	49.5
	Ciprofloxacin	>2	>2	≤1	>2	>2	45.2
	Nitrofurantoin	≤32	≤32	≤32	>64	≤32	92.5
All *K. pneumoniae* (102)	Omadacycline	4	>32	1.5	>32	4	61.8
	Tetracycline	≤4	>8	≤4	>8	≤4	58.0
	Amoxicillin-clavulanate	<4/2	>16/8	<4/2	>16/8	<4/2	65.9
	Trimethoprim-sulfamethoxazole	>2/38	>2/38	≤2/38	>2/38	>2/38	45.5
	Ciprofloxacin	≤1	>2	≤1	>2	≤1	55.7
	Nitrofurantoin	≤32	>64	≤32	>64	≤32	55.7
Total (204)	Omadacycline	4	16	0.25	>32	4	74.5 ^b^
	Tetracycline	≤4	>8	≤4	>8	≤4	52.5
	Amoxicillin-clavulanate	8/4	16/8	<4/2	>16/8	<4/2	69.6
	Trimethoprim-sulfamethoxazole	≤2/38	>2/38	≤2/38	>2/38	≤2/38	47.5
	Ciprofloxacin	2	>2	≤1	>2	≤1	50.3
	Nitrofurantoin	64	>64	≤32	>64	≤32	74.6

S, susceptible; ESBL, extended spectrum β-lactamase. ^a^ Stated n represents number of isolates tested against omadacycline. Comparator antibiotic MIC results were not available for 23 isolates (n = 181 for comparator antibiotics). ^b^ Breakpoint of *K. pneumoniae* (≤4 µg/mL) was used for interpreting *E. coli* susceptibility.

## Data Availability

The data (MIC results) presented in this study are available on request from the corresponding author.
